# Missing the Forest for the Trees - A Rare Cause of Pleural Effusion

**DOI:** 10.7759/cureus.21265

**Published:** 2022-01-15

**Authors:** Sai Samrat Kalagiri, Kavitha Venkatnarayan, Chitra Veluthat, Rajalakshmi Tirumalae, Uma Maheswari Krishnaswamy

**Affiliations:** 1 Pulmonary Medicine, St. John's National Academy of Health Sciences, Bengaluru, IND; 2 Pathology, St. John's National Academy of Health Sciences, Bengaluru, IND

**Keywords:** malignant effusion, genetic disorder, pleural effusion, neurofibromatosis, malignant peripheral nerve sheath tumour

## Abstract

Neurofibromatosis type 1 (NF-1) is a genetic disorder associated with dermatological, musculoskeletal, and neurological features. Apart from these, knowledge of other uncommon manifestations, including intrathoracic and pulmonary involvement, is crucial for early diagnosis and treatment. These patients are predisposed to various sarcomatous and non-sarcomatous malignancies. We report the case of an elderly lady with NF-1 who presented with pleural effusion related to the genetic disorder, which was missed, and elaborate on the diagnostic workup done to reach a diagnosis.

## Introduction

Neurofibromatosis type 1 (NF-1) is a genetic syndrome characteristically identified by its involvement of the nerves, skin, bones, and eyes [1]. Thoracic manifestations of the disorder are rare and encompass a spectrum ranging from chest wall and rib deformities to parenchymal lung involvement [2]. Although benign neurofibromas are the most apparent manifestations in these patients, they are predisposed to various malignancies [3]. Malignant peripheral nerve sheath tumors are one such neoplasm that is rarely seen in the general population. It is imperative to consider this as a potential diagnosis in patients with features suggestive of NF-1 for an early diagnosis and prompt therapy, lest it may lead to misdiagnosis and undue delay in diagnosis and management of these aggressive tumors.

## Case presentation

A sixty-eight-year-old lady with no co-morbidities presented with a six-month history of progressive dyspnea and dry cough. She had been evaluated for the above complaints in another hospital, where she was diagnosed to have a left-sided pleural effusion. A therapeutic pleurocentesis was performed, and analysis of pleural fluid revealed an exudative lymphocyte-predominant effusion. Since she felt symptomatically better, the patient did not follow up for further evaluation. She had worsening dyspnea for three weeks and was initiated empirically on anti-tubercular therapy elsewhere, with which there was no symptomatic relief. At presentation to our department, she complained of dull aching chest pain on the left side and had developed orthopnea for one week. There was no history of fever, hemoptysis, paroxysmal nocturnal dyspnea, loss of weight or appetite.

**Figure 1:**
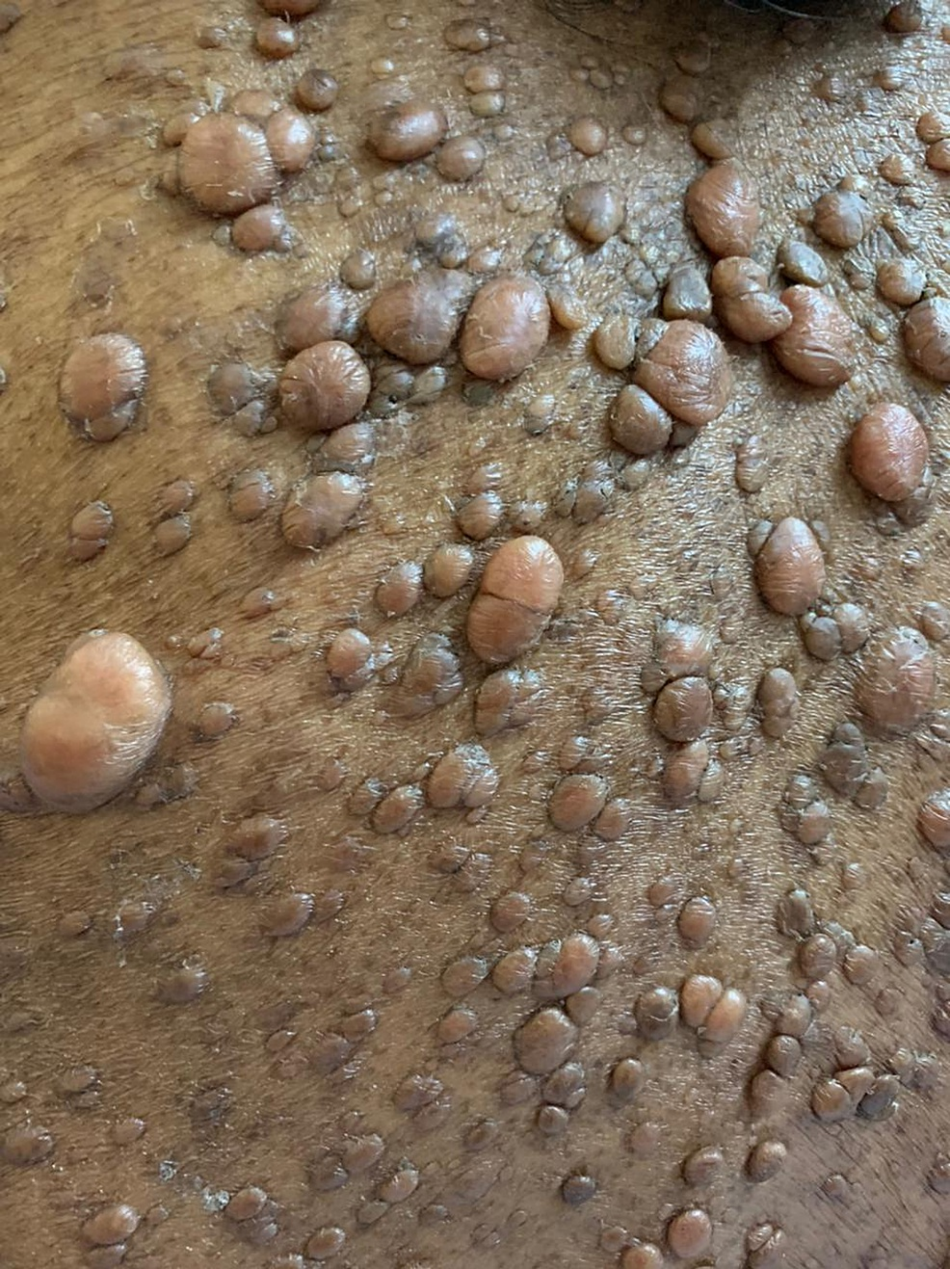
Clinical photograph showing multiple cutaneous neurofibromas on the back

Physical examination revealed a pulse rate of 98 beats/min, respiratory rate of 34 breaths/min, blood pressure of 118/70 mmHg, and an oxygen saturation of 93% on room air. Multiple varying-sized cutaneous neurofibromas were seen distributed all over the body (Figure 1). She had been diagnosed to have NF-1 at the age of 16. She had never sought medical attention after that as she was asymptomatic and did not experience any discomfort due to the skin lesions. She denied any recent increase in size or pain in any of the visible cutaneous lesions. Examination revealed a dull stony note on percussion, and absent breath sounds on auscultation in the entire left hemithorax suggestive of a massive pleural effusion. Other systems examination was unremarkable.

**Figure 2:**
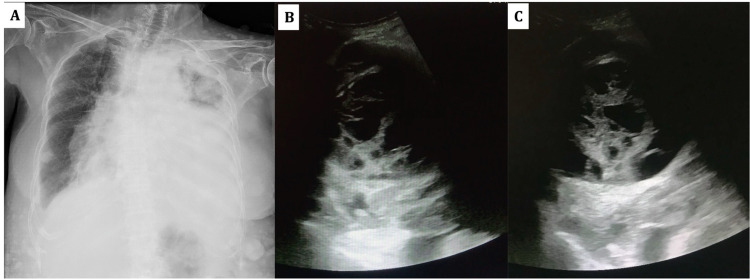
Chest radiograph and ultrasonographic image A - Chest radiograph showing left-sided pleural effusion with mediastinal shift to the opposite side and multiple subcutaneous neurofibromas; B, C - Ultrasonographic image of the left hemithorax showing effusion with multiple septations

**Figure 3:**
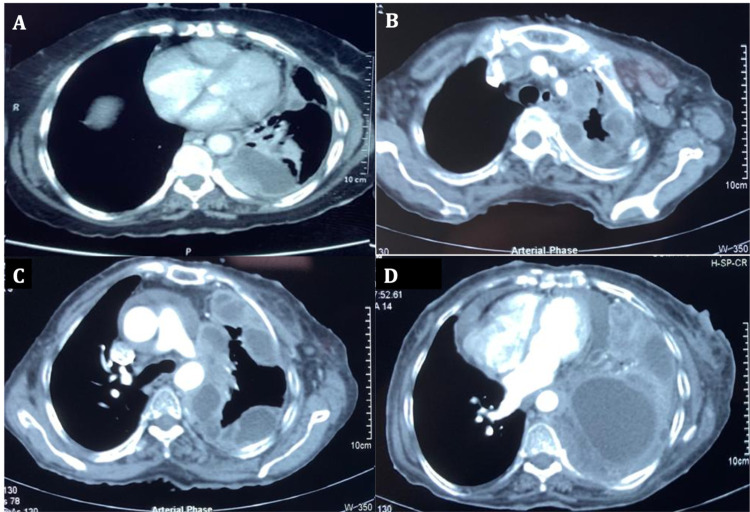
Chest CT A - CT chest performed six months prior showing left-sided minimal effusion with a pleural nodule; B, C, D - Current CT showing multiple loculations with a heterogeneous lesion involving the entire left hemithorax with mild pericardial effusion

Blood investigations, including hemogram and metabolic profile, were within normal limits. HIV serology was negative. Chest radiograph showed an opaque left hemithorax with a mediastinal shift to the opposite side (Figure 2A). Thoracic ultrasound showed left-sided massive effusion with internal septations (Figure 2B, 2C). A therapeutic thoracentesis was attempted, but minimal hemorrhagic fluid could only be aspirated. Pleural fluid analysis was suggestive of lymphocyte-predominant exudative fluid (cell count 1830/µL, neutrophils=35%, lymphocytes=65%, glucose 56 mg/dL, lactate dehydrogenase (LDH) 1254 U/L, protein 3.37 g/dL, adenosine deaminase levels 7.7 IU/L). Pleural fluid cultures were negative. Cytopathological examination showed a few atypical cells with pleomorphic nuclei, which was non-diagnostic. A chest computed tomography (CT) scan done six months back showed left-sided pleural effusion with a pleural-based nodule (Figure 3A). A repeat chest CT was done which showed a heterogeneous lesion occupying the entire left hemithorax with multiple loculations and pericardial effusion (Figure 3B, 3C, 3D). Echocardiography done for evaluation of orthopnea showed massive pericardial effusion for which pericardiocentesis was done and a pigtail was inserted. Approximately 450 ml fluid was drained over the next week, after which the pigtail was removed. Pericardial fluid was negative for malignant cells. Since thoracoscopy was technically difficult given the multiple loculations, an ultrasound-guided closed pleural biopsy was performed.

**Figure 4:**
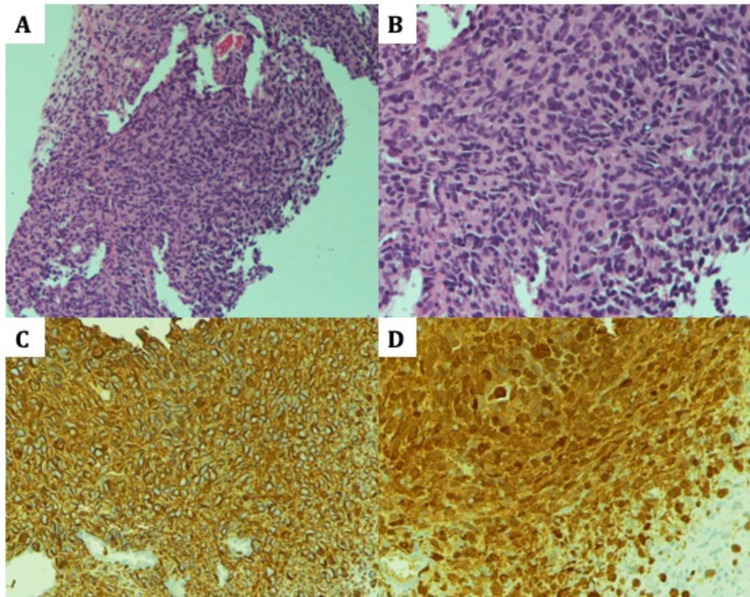
Histopathology A - Infiltrating neoplasm of spindle-shaped to plump cells in sheets (H&E, 10x); B - Neoplastic spindle cells exhibiting moderate pleomorphism and hyperchromatic nuclei (H&E, 40x); C - Cytoplasmic positivity for Vimentin (IHC-Vimentin, 20x); D - Strong nuclear and cytoplasmic positivity for S100 (IHC-S100, 40x)

Core biopsies from the pleura showed fibrocollagenous tissue with an infiltrating neoplasm composed of spindle-shaped to plump cells exhibiting moderate pleomorphism and hyperchromatic nuclei. Occasional mitotic figures were noted. There were no distinctive morphologic features. On immunohistochemistry, the neoplastic spindle cells showed strong Vimentin and focal S100 positivity and were negative for cytokeratin (CK), epithelial membrane antigen (EMA), CD34, and desmin. CK and EMA negativity excluded carcinoma, mesothelioma, and synovial sarcoma. CD34 negativity excluded solitary fibrous tumors. With the history of NF-1 and expression of S100, a neural lineage marker, a diagnosis of malignant peripheral nerve sheath tumor (MPNST) was reached (Figure 4).

Since the patient was a poor surgical candidate due to her performance status (ECOG-3), an option of palliative chemotherapy was given. However, the patient opted against palliative chemotherapy and was discharged with symptomatic treatment. With a LENT score of five, the immediate family was prognosticated regarding poor chances of survival. As further evaluation was deferred in accordance with patient preference, we could not delineate the primary lesion or presence of metastatic lesions.

## Discussion

Neurofibromatosis type 1 (NF-1) is an autosomal dominant genetic disorder caused by a mutation in the NF-1 tumor suppressor gene encoding the protein neurofibromin, predominantly expressed in the neurons and Schwann cells. The mutation is acquired de novo in more than half of these patients with no family history [1]. Also known as von Recklinghausen’s disease, it mainly presents with dermatological, musculoskeletal, neurological, and ocular manifestations. The diagnosis is based on the presence of two or more of the seven criteria defined by the National Institute of Health mentioned in Table 1 [4].

**Table 1:** NIH consensus diagnostic criteria for neurofibromatosis type 1

NIH consensus diagnostic criteria for neurofibromatosis type 1
Six or more café-au-lait spots (diameter > 5 mm before puberty and > 15 mm after puberty)
Two or more neurofibromas of any type or one plexiform neurofibroma
Axillary freckling
Two or more iris hamartomas (Lisch nodules)
Optic glioma
Typical bone lesions (sphenoid dysplasia or tibial pseudarthrosis)
One or more first-degree relatives with Neurofibromatosis type 1

Thoracic manifestations of NF-1 include chest wall involvement in the form of kyphoscoliosis, ribbon deformity of ribs, and vertebral anomalies like posterior scalloping and intrathoracic neurogenic tumors. Pulmonary involvement is rare and may manifest as interstitial lung disease, bullous or cystic lung diseases, and vascular involvement in the form of precapillary pulmonary hypertension. A combination of bullae in the upper lobes with lower lobe predominant interstitial lung disease is characteristic of NF-associated diffuse lung disease and typically occurs in the third and fourth decade [2].

Patients with NF-1 are predisposed to develop tumors, neurofibromas being the commonest of them. Neurofibromas are nerve sheath tumors comprising of Schwann cells and can be either cutaneous, subcutaneous, or plexiform. Plexiform neurofibromas arise from multiple nerve fascicles, grow along the length of the nerve and carry an increased risk of malignant transformation into MPNST. In addition to MPNST, patients with NF-1 are at increased risk for developing other sarcomatous tumors like rhabdomyosarcomas and gastrointestinal stromal tumors [3]. NF-1 is also associated with an increased risk of other non-sarcomatous malignancies, including thyroid, breast, lung, gastrointestinal, and lymphomatous malignancies [5].

MPNSTs, previously called neurofibrosarcoma or malignant schwannoma, are malignant tumors arising from a peripheral nerve or showing nerve sheath differentiation. They are rarely reported in the general population, with an incidence rate of 0.001%. NF-1 is a major risk factor associated with an increased incidence of 2-5% and accounts for nearly half of the cases [1]. History of therapeutic irradiation is the other well-established risk factor. Usually, MPNSTs arise from the neural plexus in the head and neck, extremities, and trunk. New-onset pain and an increase in the size of a pre-existing neurofibroma with or without neuro-deficits are the most common presenting symptoms [6]. 

Sporadically, MPNSTs may arise from internal organs like the retroperitoneum, abdomen, pelvis, and thorax. Thoracic MPNSTs may arise from the mediastinum, lung, pleura, or the chest wall. Identifying the origin of the tumor was difficult in our patient owing to the huge size of the lesion. The presence of an elongated mass along the course of a nerve should raise suspicion of MPNST. In patients with NF-1, any intrathoracic mass should be further evaluated for MPNST.

Radiological differentiation of benign and malignant nerve sheath tumors is difficult. A rapid increase in size, presence of high attenuation and necrosis or hemorrhage on CT, and heterogeneity on magnetic resonance imaging point towards a malignant tumor [7]. Positron emission tomography (PET) scans may be useful in differentiating a malignant tumor from a benign neurofibroma. MPNSTs are F-18 fluorodeoxyglucose (FDG) avid lesions, and a standard uptake value (SUVmax) ≥3.5 is considered to be potentially malignant, whereas a value <2.5 is likely benign. PET scan has a sensitivity of 89% to 100% and a specificity ranging from 72% to 100% to identify malignant lesions [8].

On histopathology, MPNSTs are high-grade sarcomas, with closely packed spindle cells with a high mitotic rate and necrosis. Some tumors can have epithelioid morphology with plump cells. Immunohistochemistry is essential but can be non-specific. Two-thirds of cases are S100 negative. S100 expression can also be seen in synovial sarcoma and melanoma. MPNST can also show heterologous differentiation, such as skeletal muscle (malignant triton tumor) or cartilage [9].

Multimodality treatment is usually preferred, given the aggressive nature of the tumor. Wide excision to achieve a negative tumor margin with adjuvant radiotherapy and chemotherapy consisting of an anthracycline-ifosfamide combination is preferred [10]. A tumor size of >5cm, presence of NF-1, prior irradiation, surgical resection margin status, and histological grade of the tumor have prognostic significance [11].

## Conclusions

Patients with NF-1 are predisposed to various sarcomatous and non-sarcomatous malignancies and need to be diligently followed up. MPNST is an aggressive sarcoma arising from peripheral nerves. They may appear de novo or result from the malignant transformation of a pre-existing neurofibroma. Although intrathoracic MPNSTs are rare, they should be considered in patients with NF-1 with intrathoracic masses.
